# Gangrenous small bowel obstruction resulting from appendico-ileal knotting: a rare case report from Bete Markos Medical and Surgical Center, Ethiopia

**DOI:** 10.1093/jscr/rjaf539

**Published:** 2025-07-17

**Authors:** Berhanu Kassahun, Abebe Dilie Afenigus

**Affiliations:** Department of Surgery, College of Medicine and Health Sciences, Debre Markos University, Debre Markos, PO Box 269, Gojjam, Ethiopia; Department of Nursing, College of Medicine and Health Sciences, Debre Markos University, Debre Markos, PO Box 269, Gojjam, Ethiopia

**Keywords:** gangrenous small bowel, obstruction, appendico-ileal knot, case report

## Abstract

Appendico-ileal knotting (AIK) is a rare and often overlooked cause of small bowel obstruction (SBO), frequently mimicking conditions like perforated appendicitis. We report a case of a 28-year-old Ethiopian male presenting with acute lower abdominal pain, nausea, anorexia, and bilious vomiting. Imaging suggested SBO, and emergency laparotomy revealed 1.5 l of hemorrhagic ascites and a gangrenous distal ileum caused by a 20 cm gangrenous appendix wrapped around the ileum. Surgical treatment involved untwisting the knot, appendectomy, resection of 1 m of ileum, and ileo-transverse anastomosis. The patient recovered well and was discharged on postoperative Day 6. AIK should be considered in SBO cases without previous abdominal surgery, and early surgical intervention is crucial to avoid complications.

## Introduction

Small bowel obstruction (SBO) is a common surgical emergency, usually caused by adhesions, hernias, or malignancy [1]. Rare causes like appendico-ileal knotting (AIK), involving a mobile appendix encircling the ileum, are infrequently reported and often missed preoperatively [2, 3]. AIK leads to closed-loop obstruction that can rapidly cause ischemia and gangrene if untreated [4, 5]. Due to overlapping features with more common conditions like perforated appendicitis or generalized peritonitis, AIK is diagnostically challenging, especially in low-resource settings without advanced imaging, and should be considered in patients with features of obstruction and appendicitis [6]. This report presents an intraoperatively diagnosed AIK case, emphasizing early surgical intervention.

## Methods

This case report has been reported in line with 2023 SCARE criteria [7].

### Patient information

A 28-year-old Ethiopian male with no significant past medical or surgical history presented to the emergency department with a 2-day history of progressively worsening lower abdominal pain, mild distension, anorexia, nausea, and four episodes of bilious vomiting. The pain was continuous and localized below the umbilicus. He denied fever, diarrhea, constipation, urinary symptoms, or prior abdominal trauma. There was no history of chronic illness, tuberculosis, radiation exposure, or family history of gastrointestinal disease. The patient was not on any regular medications and had no known drug allergies.

### Clinical findings

On examination, the patient appeared acutely ill and in pain. Vital signs were notable for a pulse rate of 110 bpm, respiratory rate of 22/min, SpO_₂_ of 95%, blood pressure of 130/70 mmHg, and a temperature of 36.6°C; weight was 59 kg. Conjunctivae were pink, sclerae non-icteric, and buccal mucosa moist. No abnormalities were detected in the lymphatic, chest, cardiovascular, genitourinary, musculoskeletal, integumentary, or nervous systems. Abdominal exam revealed mild distension below the umbilicus without visible peristalsis. There was direct and rebound tenderness on palpation, tympanic percussion, and hyperactive bowel sounds. Digital rectal examination was normal (Table 1).

**Table 1:** Summarizing the clinical presentation, diagnostic work-up, surgical findings, and outcomes based on the case report.

**Category**	**Details**
Clinical presentation	28-year-old Ethiopian male 2-day history of lower abdominal pain, nausea, anorexia Multiple episodes of bilious vomiting No fever, chills, or changes in bowel habits No prior abdominal surgery or trauma
Physical examination	Acutely ill appearance, visible distress Tachycardia (110 bpm), RR 22/min Localized tenderness and rebound below umbilicus Mild abdominal distension Hyperactive bowel sounds Normal digital rectal exam
Laboratory findings	WBC count: 11 000/mm^3^ (mild leukocytosis) Other labs (electrolytes, renal, liver) unavailable
Imaging studies	Abdominal X-ray: Multiple air-fluid levels consistent with SBO Ultrasound: Pelvic hyperechoic fluid collection without septation
Preoperative diagnosis	Pelvic peritonitis secondary to perforated viscus or appendicitis
Intraoperative findings	Approximately 1.5 l hemorrhagic ascitic fluid 20 cm gangrenous appendix tightly encircling distal ileum Closed-loop obstruction causing gangrene of ~1 m of distal ileum No tumors, fecaliths, or other masses
Surgical intervention	Untwisting of appendico-ileal knot Appendectomy Resection of gangrenous ileum End-to-side ileo-transverse anastomosis
Postoperative course	Uneventful recovery Tolerated oral intake by postoperative Day 4 Discharged on postoperative Day 6 Follow-up at Day 11 showed full recovery with normal bowel function

### Diagnostic assessment

Laboratory evaluation showed a white blood cell count of 11 000/mm^3^; differential counts (lymphocytes, neutrophils, basophils) were not documented. Imaging studies included a plain abdominal X-ray, which revealed multiple air-fluid levels consistent with SBO. Abdominopelvic ultrasound showed a hyperechoic pelvic fluid collection without septation or echogenic debris, suggestive of inflammatory, or hemorrhagic fluid.

### Diagnosis

Given the combination of clinical, laboratory, and imaging findings, a preoperative diagnosis of pelvic peritonitis likely secondary to viscus perforation or perforated appendicitis was established. However, due to diagnostic uncertainty and progressive symptoms, the patient was taken for emergency exploratory laparotomy. Intraoperatively, the diagnosis was revised to a gangrenous SBO caused by AIK, a rare surgical entity.

### Therapeutic intervention

**Figure 1:**
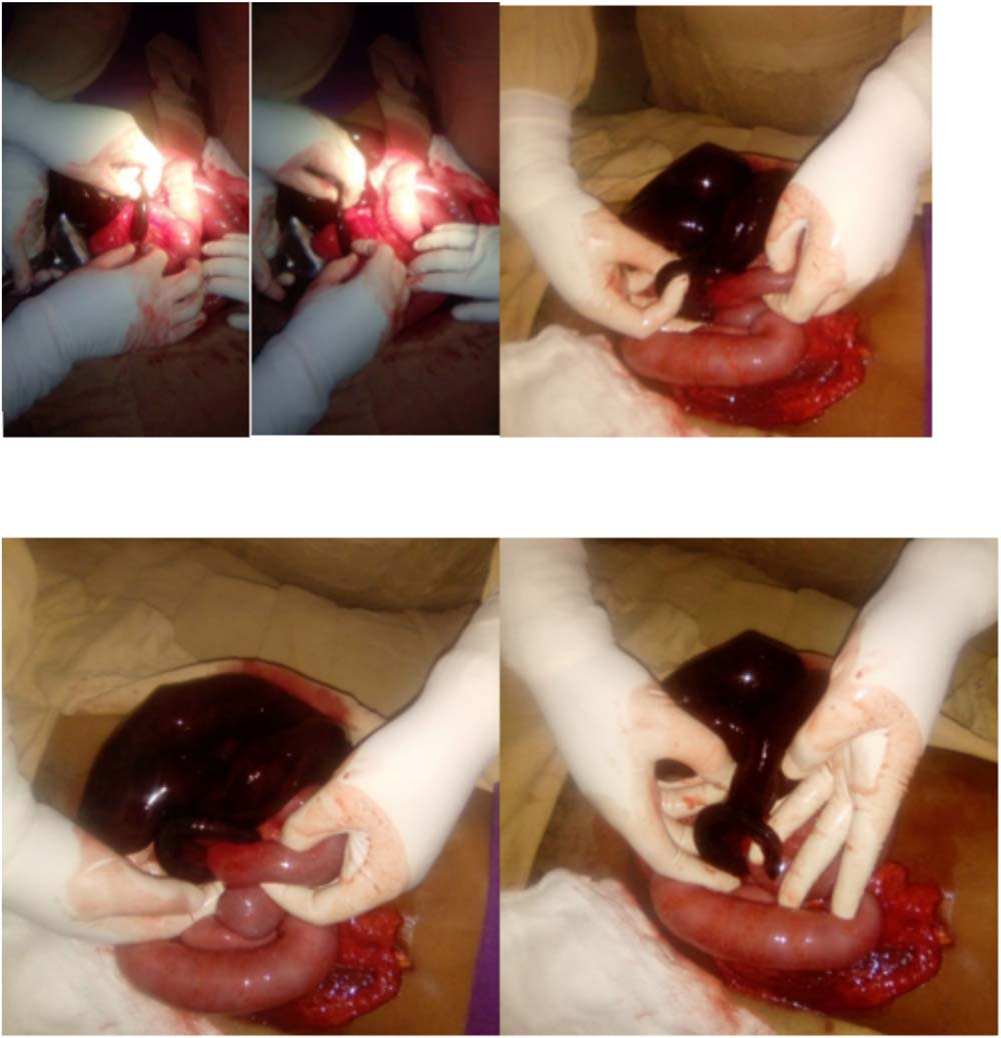
Intraoperative photo showing appendiceal knot around distal ileum.

An emergency exploratory laparotomy was performed under general anesthesia. Intraoperatively, ~1.5 l of hemorrhagic ascitic fluid was encountered and suctioned. A 20-cm-long gangrenous appendix was found tightly encircling a loop of the distal ileum, forming a constrictive knot. This resulted in a closed-loop obstruction and gangrene involving ~1 m of the distal ileum. The knot was carefully untwisted, and an appendectomy was performed. The 5-cm distal ileal stump was closed, and an end-to-side anastomosis was performed between the proximal ileum and the transverse colon (Fig. 1). The procedure was completed successfully without intraoperative complications.

### Follow-up and outcomes

Postoperatively, the patient received intravenous antibiotics, analgesics, and crystalloids. Recovery was uneventful, with no signs of infection, anastomotic leak, or ileus. He tolerated oral intake by postoperative Day 4 and was discharged on Day 6. At follow-up on Day 11 (5 days post-discharge), he was asymptomatic, with normal bowel function, no complications, and had resumed a regular diet, confirming complete clinical recovery.

## Discussion

AIK is a rare cause of SBO [8]. It results from a long, mobile appendix encircling the ileum, and leading to strangulation and ischemia [9]. Preoperative diagnosis is challenging due to nonspecific symptoms and limited imaging capabilities, especially in resource-limited settings [10].

SBO is typically diagnosed based on radiologic findings and clinical features like colicky abdominal pain, early bilious vomiting, central distension, and late constipation [11]. Preoperative diagnosis of AIK is difficult due to its rarity and inconsistent detection on X-rays or ultrasound [12]. In our case, the patient presented with typical SBO symptoms and was diagnosed intraoperatively. Management involves prompt laparotomy, detorsion, appendectomy, bowel resection, and appropriate anastomosis or stoma formation. Early surgical intervention is essential to prevent complications like gangrene or perforation.

Awareness of AIK is crucial when evaluating SBO in patients without prior surgery or hernia [2], and this case emphasizes the importance of clinical judgment when imaging is inconclusive.

## Conclusion

This case report educates clinicians on the rare but serious entity of AIK as a cause of SBO. High clinical suspicion and timely exploratory laparotomy are essential in ambiguous presentations, especially where advanced diagnostics are unavailable. Early surgical management prevents severe complications and ensures good outcomes.

### What is already known on this topic

- SBO is commonly caused by adhesions, hernias, or tumors.
- AIK is a rare cause of SBO that can cause rapid bowel ischemia.
- Preoperative diagnosis is difficult due to nonspecific symptoms and limited imaging findings.

### What this study adds

Highlights AIK in a young adult without previous abdominal surgery.

- Emphasizes the importance of early surgical exploration in resource-limited settings.
- Demonstrates successful management with appendectomy and ileal resection leading to full recovery.

## Patient perspective

The patient expressed gratitude for the prompt surgical care and reported full satisfaction with the outcome during the follow-up visit.
